# Identification of an IL-4-Related Gene Risk Signature for Malignancy, Prognosis and Immune Phenotype Prediction in Glioma

**DOI:** 10.3390/brainsci12020181

**Published:** 2022-01-29

**Authors:** Ying Qi, Xinyu Yang, Chunxia Ji, Chao Tang, Liqian Xie

**Affiliations:** 1 Department of Neurosurgery, Huashan Hospital, Shanghai Medical College, Fudan University, Shanghai 200040, China; 18211220054@fudan.edu.cn (Y.Q.); 20211220056@fudan.edu.cn (X.Y.); 13211010007@fudan.edu.cn (C.J.); chaotang@fudan.edu.cn (C.T.); 2 Neurosurgical Institute of Fudan University, Fudan University, Shanghai 200040, China

**Keywords:** glioma, IL-4, 10-gene signature, prognosis, microenvironment

## Abstract

Background: Emerging molecular and genetic biomarkers have been introduced to classify gliomas in the past decades. Here, we introduced a risk signature based on the cellular response to the IL-4 gene set through Least Absolute Shrinkage and Selection Operator (LASSO) regression analysis. Methods: In this study, we provide a bioinformatic profiling of our risk signature for the malignancy, prognosis and immune phenotype of glioma. A cohort of 325 patients with whole genome RNA-seq expression data from the Chinese Glioma Genome Atlas (CGGA) dataset was used as the training set, while another cohort of 667 patients from The Cancer Genome Atlas (TCGA) dataset was used as the validating set. The LASSO model identified a 10-gene signature which was considered as the optimal model. Results: The signature was confirmed to be a good predictor of clinical and molecular features involved in the malignancy of gliomas. We also identified that our risk signature could serve as an independently prognostic biomarker in patients with gliomas (*p* < 0.0001). Correlation analysis showed that our risk signature was strongly correlated with the Tregs, M0 macrophages and NK cells infiltrated in the microenvironment of glioma, which might be a supplement to the existing incomplete innate immune mechanism of glioma phenotypes. Conclusions: Our IL-4-related gene signature was associated with more aggressive and immunosuppressive phenotypes of gliomas. The risk score could predict prognosis independently in glioma, which might provide a new insight for understanding the IL-4 involved mechanism of gliomas.

## 1. Introduction

Gliomas are the most prevalent and aggressive brain tumors, with extremely poor prognosis in adults. Among all grades of gliomas, glioblastoma (GBM) is the most devastating type with a median overall survival time of approximately 19 months [1]. At recurrence, patients always have a very poor survival rate despite the beneficial treatments including second surgery [1] and re-irradiation [2]. In the past decade, newly emerging therapeutic approaches such as tumor-treating fields (TTF) and several immunotherapies were introduced in the hopes of GBM treatment [3]. However, the majority of the immunotherapies including PD-1/PD-L1 checkpoint inhibitors, chimeric antigen receptor-T cells (CAR-T), and adoptive T cell strategies ended in the failure of GBM treatments [4,5,6]. These failures strongly indicated that beyond the T cell-based adaptive immunity, innate immunity might be one of the most critical aspects to regulate anti-tumor immunity in the glioma microenvironment [4]. Many works have focused on the microglia, while other innate immune cells such as infiltrating macrophages and NK cells are becoming more attractive in the studies of the GBM immune microenvironment [7,8]. Despite the existing efforts in glioma research, little progress has been made in understanding the molecular mechanism of gliomas, and the effects of innate immunity in the glioma microenvironment still remain incomplete [9].

Interleukin-4 (IL-4), a Th2 cytokine mainly produced by activated T cells and mast cells, is confirmed to regulate the proliferation of lymphocytes [10]. In addition to its immune function, IL-4 produced by cancer cells is also reported to promote tumor proliferation and aggressiveness in glioma, bladder cancer, breast cancer and other epithelial tumors through STAT6 signal transduction pathways [11,12]. Enhanced IL-4 secretion of cancer cells could also be involved in tumor-associated macrophages (TAM)-induced tumor growth and metastasis [13]. Polymorphisms in the IL-4 receptor genes are reported to influence the glioma survival, which indicate that IL-4-induced inflammatory pathways might regulate the glioma development and prognosis [14]. However, the role of cellular response to IL-4 in glioma development remains unclear.

In our study, we focus on the cellular response to the IL-4 gene set from gene ontology in gliomas. Consensus clustering was firstly applied to identify that the cellular response to the IL-4 gene set had the ability to distinguish clinicopathological features of gliomas. Next, a cellular response to IL-4-related gene risk signature was generated in the CGGA dataset through LASSO regression, and further validated in the TCGA dataset. Our risk signature was observed to be strongly associated with clinicopathological features of gliomas and to be an independent prognostic factor for both all grade gliomas and GBM. In addition, we found that this cellular response to the IL-4-related gene risk signature was closely related to tumor infiltrating lymphocytes (including M0 macrophages, NK cells, and Tregs) in the glioma microenvironment, which indicated a potential association between the cellular response to IL-4 and the immune phenotype of gliomas. We believe that our bioinformatic analysis might provide a new insight for understanding the IL-4 involved mechanism of gliomas.

## 2. Materials and Methods

### 2.1. Data Collection

Two population datasets were analyzed in this study: a glioma dataset from the Chinese Glioma Genome Atlas (CGGA) dataset and a glioma dataset from The Cancer Genome Atlas (TCGA) dataset. The RNA-seq expression data and clinical information of 325 glioma patients from CGGA dataset (http://www.cgga.org.cn, 1 October 2020) was used as the training set [15,16]. RNA-seq data and clinical information of 667 glioma patients from TCGA dataset (http://cancergenome.nih.gov, 1 October 2020) was used as validation set [17,18]. 

### 2.2. Consensus Clustering

All 28 genes in the cellular response to IL-4-related gene set were extracted from Molecular Signatures Database v6.2 [15]. Determined by the median absolute deviation (MAD), the most variable genes of the cellular response to IL-4-related gene set were selected for the consensus clustering analysis through ConsensusClusterPlus package [19]. R programming language was used for consensus clustering for detecting the cellular response to IL-4-related glioma subgroups of the CGGA training set. The optimal number of the clusters was further determined by quantitative stability evidence in an unsupervised analysis.

### 2.3. Construction of the Gene Risk Signature

Screened by univariate Cox regression analysis in the CGGA training set, all genes with high prognostic value (*p* < 0.05) in the cellular response to IL-4-related gene set were selected for Least Absolute Shrinkage and Selection Operator (LASSO) regression analysis [20]. We used glmnet package in R programming language as our LASSO regression tool. The generalized linear model produced by LASSO regression analysis was further analyzed with 10-fold cross validation in order to generate the minimum cross validated error [21]. Based on the minimum cross validated error, expressions (expr) of 10 genes in the cellular response to IL-4-related gene set and their regression coefficients (Coef) were eventually achieved. Then, the risk score for each patient in the CGGA training set and TCGA validation set was calculated by the following formula: risk score = exprgene1 × Coefgene1 + exprgene2 × Coefgene2 + … + exprgene10 × Coefgene10.

All patients in the CGGA training set and TCGA validation set were then separated into high or low risk group according to the median risk score cutoff. Survival analysis based on the risk score was evaluated by Kaplan–Meier survival curve using R programming language. Univariate and multivariate survival analysis was performed by using Cox proportional hazards model in R programming language.

### 2.4. Estimation of the Abundances of Immune Cell Types

For evaluating the association between the cellular response to IL-4-related gene risk signature and the immune phenotype of glioma, estimation of the abundances of immune cell types through gene expression data in CGGA and TCGA datasets was achieved by CIBERSORT package in R programming language. We used LM22 introduced by Newman, A.M. et al. as our input marker matrix of 22 types of immune cells [22]. The correlation between our risk signature and immune cell was validated by corrplot package in R programming language, and all the heatmaps were produced through ComplexHeatmap package in R programming language [23].

### 2.5. Statistical Analyses

Main statistical analysis including Student’s *t*-test, chi-square test, and Pearson’s test were also performed in R programming language. Statistical significance was considered at the level of *p* < 0.05.

## 3. Results

### 3.1. Classification of Gliomas Based on Cellular Response to IL-4-Related Gene Set

**Figure 1:**
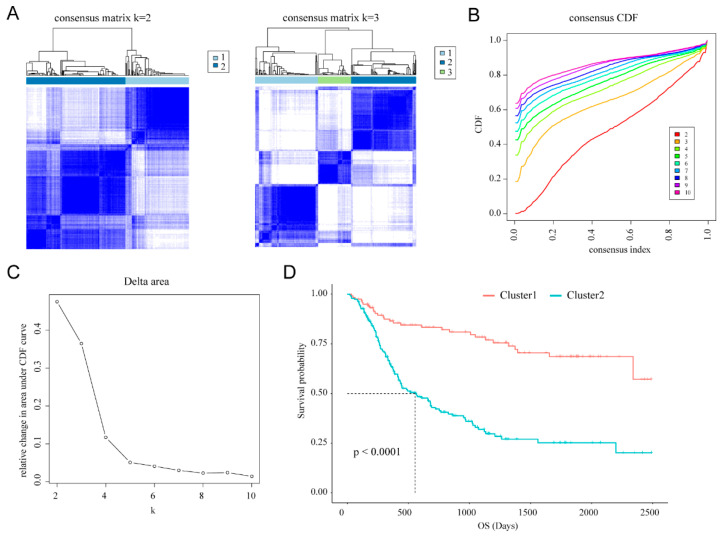
Classification of gliomas based on the cellular response to IL-4-related gene set in CGGA dataset. (**A**) Consensus clustering matrix of 325 CGGA samples for k = 2 and k = 3. (**B**) Consensus clustering CDF for k = 2 to k = 10. (**C**) Relative change in area under CDF curve for k = 2 to k = 10. (**D**) Survival analysis using Kaplan–Meier method for two clusters.

The gene expression profiling of all genes in the cellular response to IL-4-related gene set obtained from the CGGA training set was used as variables of consensus clustering. The result of consensus clustering indicated that 325 patients in the training set could be classified into two robust clusters with clustering stability increasing between k = 2 to k = 10 (Figure 1A–C; Supplementary Figure S1). Kaplan–Meier survival analysis showed that patients with gliomas in cluster2 (*n* = 201) had a significantly poorer prognosis than in cluster1 (*n* = 124; median OS: 555 vs. 1082 days; Figure 1D). Furthermore, differences in clinicopathological features between these two clusters were also found through Student’s *t*-test and chi-square test (Supplementary Table S1). Cluster2 had a strong correlation with older age at diagnosis (median age: 46, *p* < 0.001), classical or mesenchymal subtypes (62.19%, *p* < 0.001), glioblastoma phenotype (58.21%, *p* < 0.001), IDH wildtype (70.65%, *p* < 0.001), and 1p/19q non-codeletion (82.59%, *p* < 0.001). By contrast, cluster1 mainly represented younger age at diagnosis (median age: 38, *p* < 0.001), proneural or neural subtypes (86.29%, *p* < 0.001), lower grade phenotype (78.23%, *p* < 0.001), and IDH mutation (87.09%, *p* < 0.001). Our results indicated that cellular response to IL-4-related gene set was involved in the malignancy of gliomas and strongly correlated to prognosis.

### 3.2. Identification of a 10-Gene Risk Signature Associated with Cellular Response to IL-4

**Figure 2:**
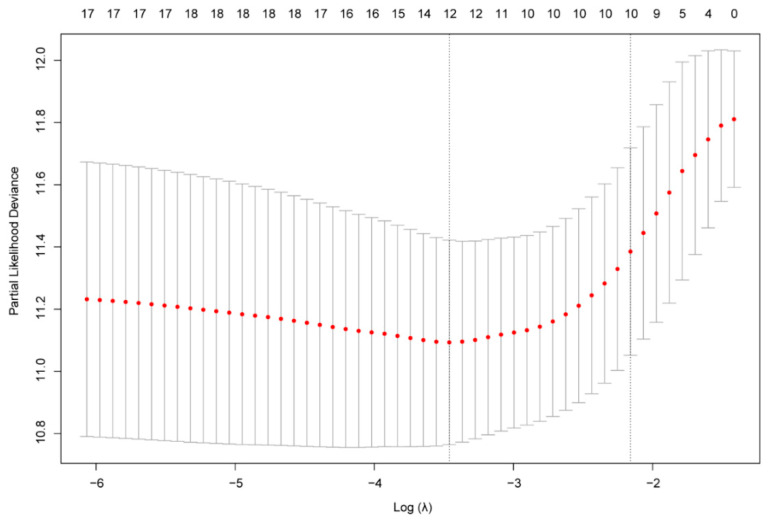
Lasso regression analysis of IL-4-associated genes with high prognostic values (Uni-Cox *p* < 0.05) generated 10 genes as covariates to calculate the gene risk signature. Dotted line indicated the most preferred λ value as the L1-regularization of Lasso regression. Box lines represented the variances of partial likelihood deviance of the λ value.

Through univariate Cox regression analysis, all cellular responses to IL-4-related genes with high prognostic values (*p* < 0.05) were selected for further analysis in the CGGA training set. To identify the risk signature associated with cellular response to IL-4, genes with high prognostic values further underwent the LASSO regression analysis. After 10-fold cross validation, LASSO regression analysis generated 10 genes (CORO1A, FASN, HSPA5, IL2RG, LEF1, MCM2, NFIL3, PML, RPL3, TUBA1B) in total as active covariates to calculate the risk score (Figure 2; Table 1). The signature risk score of each patient in the training set and validating set was then calculated with the LASSO regression coefficients and expression value of these 10 genes through equations.

### 3.3. Cellular Response to IL-4-Related Gene Risk Signature Distinguished the Clinicopatho Logical Features of Gliomas

**Figure 3:**
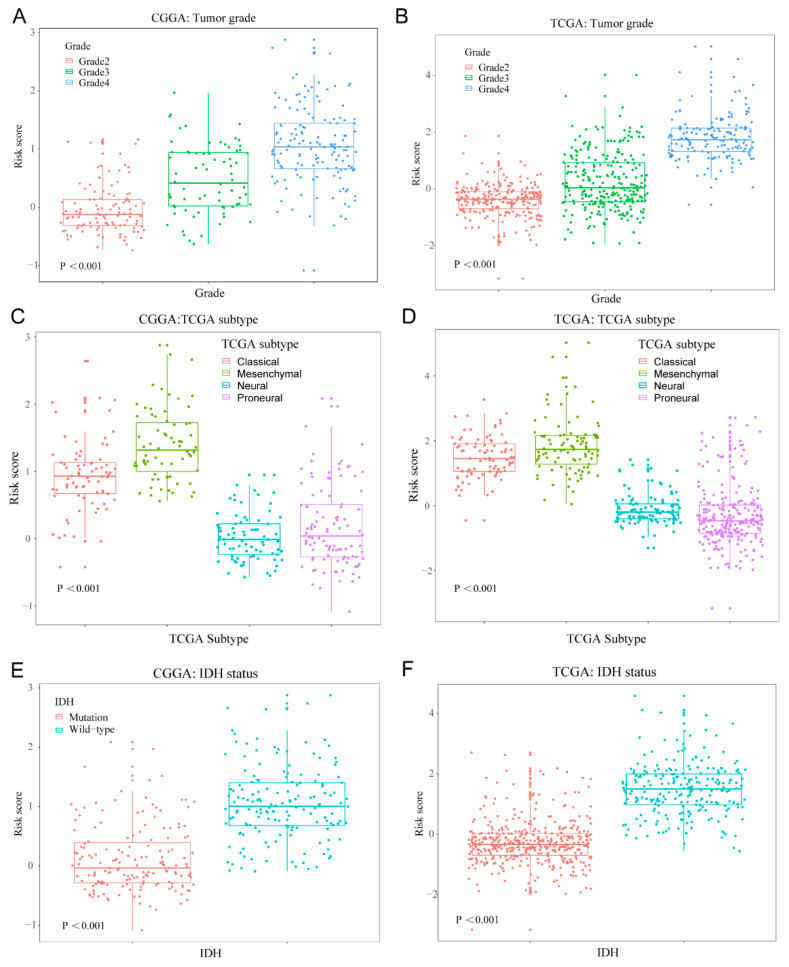
The 10-gene risk signature distinguished the clinicopathological features of gliomas. Distribution of the 8-gene risk signature with different tumor grades (**A**,**B**), TCGA subtypes (**C**,**D**) and IDH status (**E**,**F**).

After calculating the 10-gene risk signature score of each patient, we observed that higher risk scores were found in glioblastoma than lower grade gliomas (*p* < 0.001), in classical and mesenchymal subtypes than other subtypes (*p* < 0.001), and in the IDH wildtype than the IDH mutation (*p* < 0.001) in the CGGA dataset (Figure 3A,C,E). A similar distributional pattern of the risk score was also observed in the TCGA dataset (Figure 3B,D,F). Then, we classified the patients in the training set into high-risk group and low-risk group by using median signature risk score as the cutoff value. Patients in the high-risk group were linked to older age at diagnosis (median age: 46.5, *p* < 0.001), classical or mesenchymal subtypes (79.01%, *p* < 0.001), glioblastoma phenotype (70.98%, *p* < 0.001), IDH wildtype (78.39%, *p* < 0.001), and 1p/19q non-codeletion (95.23%, *p* < 0.001, Supplementary Table S2). By contrast, patients in the low-risk group were associated with younger age at diagnosis (median age: 39, *p* < 0.001), proneural or neural subtypes (91.41%, *p* < 0.001), lower grade phenotype (82.21%, *p* < 0.001), and IDH mutation (80.98%, *p* < 0.001; Supplementary Table S2). In the TCGA dataset, we also observed that patients in the high-risk group were correlated with older age at diagnosis (median age: 54, *p* < 0.001), classical or mesenchymal subtypes (67.91%, *p* < 0.001), IDH wildtype (69.66%, *p* < 0.001), and 1p/19q non-codeletion (96.88%, *p* < 0.001, Supplementary Table S2), while patients in the low-risk group had a strong correlation with younger age at diagnosis (median age: 39, *p* < 0.001), proneural or neural subtypes (99.62%, *p* < 0.001), lower grade phenotype (99.70%, *p* < 0.001), and IDH mutation (97.03%, *p* < 0.001; Supplementary Table S2). These results indicated that the 10-gene risk signature associated with cellular response to IL-4 could distinguish the malignancy of gliomas.

### 3.4. Prognostic Value of Cellular Response to IL-4-Related Gene Risk Signature in All Grade Gliomas and Glioblastoma

**Figure 4:**
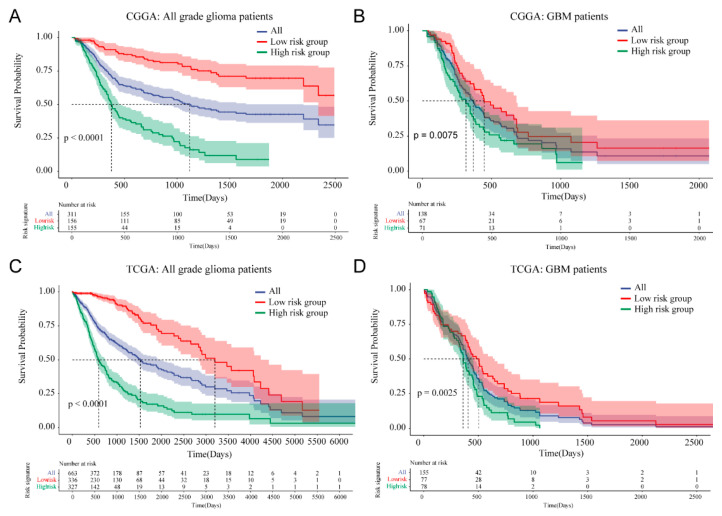
Prognostic value of 10-gene risk signature in CGGA and TCGA dataset. (**A**,**B**) Kaplan–Meier Survival curves for all grade gliomas and GBM patients in CGGA dataset. (**C**,**D**) Kaplan–Meier Survival curves for all grade gliomas and GBM patients in TCGA dataset.

In the CGGA dataset, Kaplan–Meier survival analysis revealed that patients in the high-risk group (*n* = 155) had a significantly poorer prognosis compared with patients in the low-risk group (*n* = 156; median OS: 376 days vs. NA; *p* < 0.001; Figure 4A). In the TCGA dataset, patients in the high-risk group (*n* = 327) were also found to have much shorter overall survival times than patients in the low-risk group (*n* = 338, median OS: 592 vs. 3200 days; *p* < 0.001; Figure 4C). After taking important clinical and molecular factors (including age, gender, WHO grade, IDH status, chemotherapy and radiotherapy) into account, univariate and multivariate Cox analysis further demonstrated that this risk score was an independent prognostic factor of prognosis in the CGGA dataset (Table 2). Cox proportional hazard model also found risk score could serve as an independent prognostic factor in the TCGA dataset (Table 2). When focusing on the GBM phenotype, we also observed that patients in the high-risk group (*n* = 71) had a shorter OS than patients in the low-risk group (*n* = 67) of the GBM phenotype in the CGGA dataset (median OS: 315 vs. 447 days; *p* = 0.0075; Figure 4B). Results in the TCGA dataset further validated the prognostic value of the risk signature in the GBM phenotype (median OS: 360 vs. 505 days; *p* = 0.0025; Figure 4D). These results indicated that our 10-gene risk signature associated with cellular response to IL-4 had high prognostic value in both all grade gliomas and glioblastoma.

### 3.5. Cellular Response to IL-4-Related Gene Risk Signature Was Correlated with Inhibited Immune Phenotype of Gliomas

**Figure 5:**
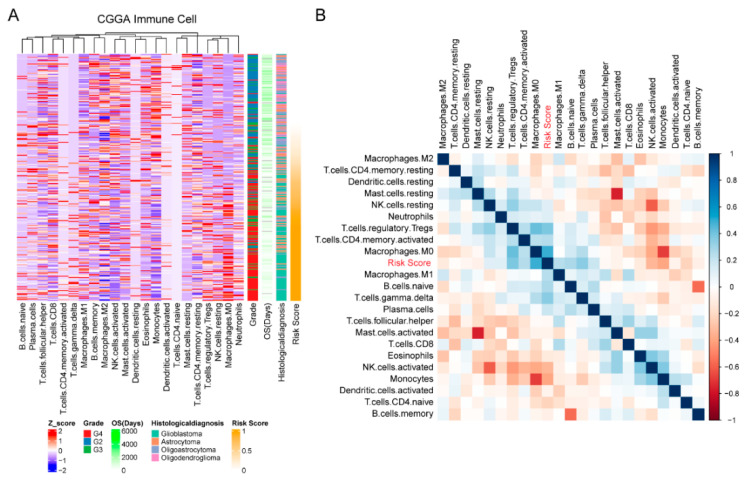
The 10-gene risk signature distinguished different local immune states in gliomas. (**A**) Analysis of heat map in CGGA dataset. (**B**) Correlation analysis in CGGA dataset.

**Figure 6:**
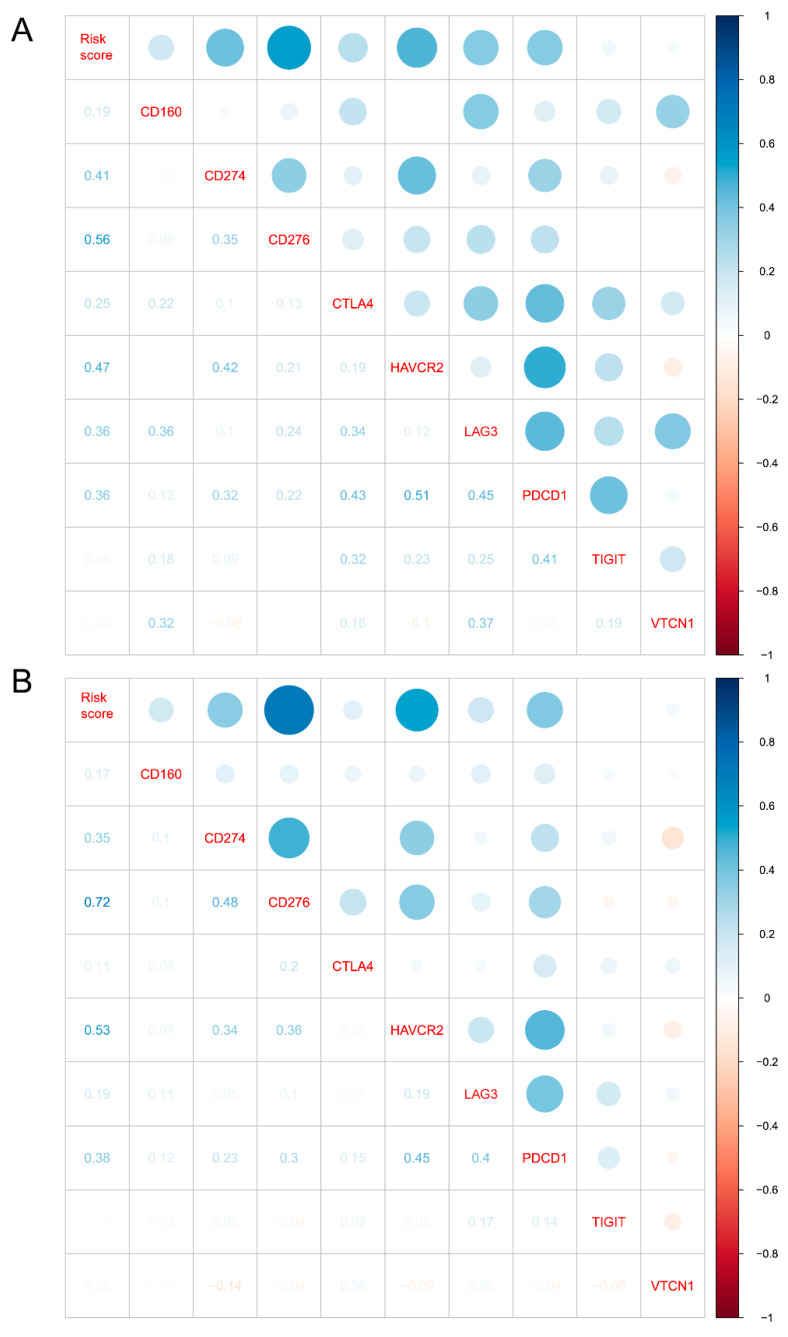
The 10-gene risk signature was strongly correlated with inhibited immune phenotype of gliomas. (**A**) Correlation analysis between risk score and immune suppressor in CGGA dataset. (**B**) Correlation analysis between risk score and immune suppressor in TCGA dataset.

After confirming the clustering and prognostic value of our IL-4-related gene risk signature, we then investigated the potential role of our risk signature in the immune phenotype of gliomas. We firstly calculated the abundances of 22 immune cell types in both the CGGA and TCGA datasets through the CIBERSORT package, and then presented the correlation between immune cells and our risk signature through heatmaps. In the CGGA datasets, higher risk score was strongly associated with less NK cells, less monocytes, less mast cells, more Tregs and more M0 macrophages (Figure 5). A similar phenotype was seen in the TCGA datasets, with higher risk score associated with less NK cells, less monocytes, less mast cells, more Tregs and more M0 macrophages (Supplementary Figure S2). With Pearson’s test, our risk score was found to be strongly correlated with immunosuppressive factors (Figure 6), including CD274 (PD-L1), PDCD1 (PD-1), CTLA4, LAG3, HAVCR2 (TIM3), and so on. These results indicated that our cellular response to IL-4-related risk signature might be correlated with the inhibited immune phenotype of gliomas.

## 4. Discussion

Gliomas are the most frequent tumors of the central nervous system, with extremely poor prognosis in adults. In 2016, the WHO classification of CNS tumors was revised for the first time using molecular and genetic biomarkers (IDH mutation and 1p/19q codeletion) to classify gliomas [24]. Then, the new WHO classification published in 2021 included more molecular biomarkers [25]. Since then, a diverse set of biomarkers have been implicated as prognostic indicators in gliomas, including checkpoint molecules (PD-1, CTLA-4, TIM-3) [26,27,28], growth/angiogenesis proteins (EGFR) [29], and cytokines (TGF-β, IL-4, IL-13) [30,31]. Among them, the effects of IL-4/IL-4 receptors were investigated in the last decade [32]. Some research advocated IL-4/IL-4 receptors could serve as excellent biomarkers and immunotherapeutic targets [33], while others found polymorphisms in IL-4 genes were not associated with glioma risk independently [32].

With the development of bioinformatic technologies, gene risk signature analysis emerged as a useful method to identify prognostic signatures in almost all kinds of cancers [34]. In gliomas, gene risk signature analysis also identified several new biomarkers including immune-related, metabolism-related, and inflammation-related gene signatures [15,35,36,37]. Here, we introduced a cellular response to IL-4-related gene signature as a newly discovered biomarker of clinicopathological features, prognosis, and immune phenotype of gliomas. We firstly confirmed that 28 genes on the cellular response to IL-4 pathway had the ability to distinguish the key clinicopathological features of gliomas in both CGGA and TCGA datasets. Then, we built the cellular response to IL-4 gene risk signature through LASSO regression analysis. Our cellular response to IL-4 gene risk signature was found to be strongly correlated with previously confirmed clinical features of gliomas including WHO grade, molecular subtypes, 1p/19q codeletion status and IDH status [24]. Using our gene risk signature, patients with higher risk score tend to be associated with the higher WHO glioma grade, the more invasive molecular subtypes (classical and mesenchymal), 1p/19q non-codeletion and IDH wide type, which represented worse prognosis. By contrast, the lower WHO glioma grade, the less invasive TCGA subtypes (proneural and neural subtype) and IDH mutation were preferentially associated with patients in the lower risk group. Our IL-4-related signature can work as a molecular biomarker to classify glioma patients combined with current genetic biomarkers, which will be beneficial to predict survival more precisely and even can be used to predict clinical response to adjuvant therapies such as immunotherapy. By analyzing the Kaplan–Meier survival curve and Cox proportional hazards model, we found our gene risk signature could serve as an independent prognostic marker of gliomas. In both CGGA and TCGA datasets, patients in the high-risk group had a significantly poorer prognosis compared with patients in the low-risk group (CGGA, median OS: 376 days vs. NA; TCGA, median OS: 592 vs. 3200 days). Considering the GBM phenotype, patients in the high-risk group also had a significantly shorter OS than patients in the low-risk group (CGGA, median OS: 315 vs. 447 days; TCGA, median OS: 360 vs. 505 days).

Next, we focus on the relationship between our gene risk signature and immune phenotype of the gliomas by CIBERSORT algorithm. In addition to the traditional surgery, chemotherapy and radiotherapy, immunotherapy has been regarded as the next generation approach in the treatment of gliomas. A range of different immunotherapies such as PD-1/PD-L1 checkpoint inhibitors, chimeric antigen receptor-T cells (CAR-T) and adoptive T cell strategies have currently been actively investigated in patients with gliomas [4,5,6]. Unfortunately, negative outcomes of these clinical trials challenge the concept of immunotherapy as a single modality treatment of gliomas [38]. Local immunosuppression in the glioma microenvironment might be responsible for the failure of current immunotherapies. The pathologic findings of most patients with gliomas showed a typical ‘cold’ tumor microenvironment with exhausted CD8+T cells and enriched Tregs [38,39]. Our cellular response to IL-4 gene risk signature showed to be highly correlated with Tregs, and higher risk scores were associated with more infiltrating Tregs. However, our risk signature was not found to be correlated with the infiltrating CD8+T cells. In addition to the adoptive immune cells, innate immune cells such as macrophages and NK cells have been a new trend in the research of glioma immunotherapy [40]. We also calculated the correlation between our risk signature and innate immune cells and found higher risk scores were strongly associated with more M0 macrophages and less NK cells. Unlike the classically activated immunostimulant M1 phenotype macrophages and alternatively activated immunosuppressive M2 phenotype, M0 macrophages are nonpolarized macrophages and found to be positively associated with malignant phenotypes of gliomas [41]. Contrary to the traditional concept, glioma-infiltrated macrophages were recently found to resemble the M0 macrophage phenotype instead of M1 or M2 phenotype [42]. The genetic and molecular mechanism of immunosuppressive M0 macrophages in gliomas remains unclear. According to our bioinformatic analysis and current dogma that Tregs secrete IL-4 to trigger the development of tumor-associated macrophages with immunosuppressive properties [43], we considered our cellular response to IL-4-related gene signature as evidence of the immunosuppressive mechanism of M0 macrophages in the glioma microenvironment. Moreover, NK cells are known as innate immune effective cells but are frequently exhausted in the glioma microenvironment [40]. Our IL-4-related risk signature might also indicate a potential genetic pathway of NK cell exhaustion in the gliomas.

## 5. Conclusions

In summary, we provided a cellular response to the IL-4-related gene signature as an excellent clinicopathological, prognostic and immune biomarker of gliomas in this study. In the future, the pathway involved in IL-4 to regulate the infiltration of immune cells will be an intriguing topic, especially in patients with high score, which may be beneficial to develop a novel immunotherapy or clinical biomarker. We believe that our bioinformatic analysis might provide new insight for understanding the IL-4 involved mechanism of gliomas.

## Figures and Tables

**Table 1:** Univariate Cox regression analysis and LASSO regression coefficients of 10 genes generated by LASSO regression analysis.

Gene	LASSO Regression Coefficient
CORO1A	0.020692273
FASN	−0.019673763
HSPA5	0.000533637
IL2RG	0.051870857
LEF1	0.038347165
MCM2	0.059818862
NFIL3	0.016612283
PML	0.101586488
RPL3	−0.002205126
TUBA1B	0.000593799

**Table 2:** Univariate and multivariate Cox regression analysis of the clinical features and risk score for OS in CGGA and TCGA datasets.

Variables	Univariate Analysis			Multivariate Analysis		
	Hazard Ratio	95% CI	*p* Value	Hazard Ratio	95% CI	*p* Value
Training set CGGA RNA-seq cohort (*n* = 325)						
Age	1.0	1.0~1.1	<0.0001	1.01	0.99~1.03	0.22
Gender	1.2	0.83~1.7	0.37	1.32	0.88~1.96	0.176
Grade	5.9	4.1~8.6	<0.0001	1.79	1.07~3.0	0.026
IDH status	4.3	3~6.2	<0.0001	1.25	0.72~2.14	0.428
MGMT status	1.4	0.99~2.0	0.058	1.0	0.68~1.47	0.999
Chemotherapy	1.2	0.87~1.7	0.23	0.80	0.54~1.19	0.276
Radiotherapy	0.41	0.28~0.58	<0.0001	0.41	0.27~0.61	<0.001
Risk score	3.9	3.1~4.8	<0.0001	2.7	1.93~3.78	<0.001
Validation set TCGA RNA-seq cohort (*n* = 667)						
Age	1.1	1.1~1.1	<0.0001	1.03	1.02~1.04	<0.0001
Gender	1.2	0.96~1.6	0.11	1.48	1.06~2.1	0.021
Grade	9.1	6.9~12.0	<0.0001	1.63	1.04~2.6	0.033
IDH status	9.8	7.4~13.0	<0.0001	2.78	1.61~4.8	<0.001
MGMT status	3.3	2.5~4.3	<0.0001	1.19	0.81~1.7	0.367
Chemotherapy	0.41	0.27~0.61	<0.0001	0.63	0.4~1.0	0.052
Radiotherapy	2.1	1.5~2.9	<0.0001	1.05	0.63~1.7	0.843
Risk score	2.3	2.1~2.5	<0.0001	1.34	1.12~1.6	<0.033

## Data Availability

Publicly available datasets were used in this study. The datasets analyzed in the present study were retrieved from The Cancer Genome Atlas and Chinese Glioma Genome Atlas databases.

## Supplementary Materials

The following supporting information can be downloaded at: https://www.mdpi.com/article/10.3390/brainsci12020181/s1, Figure S1: Consensus clustering matrix of 325 CGGA samples for k = 4 to k = 10; Figure S2: The 10-gene risk signature distinguished different local immune states in gliomas. (A) Analysis of heat map in TCGA dataset. (B) Correlation analysis in TCGA dataset; Table S1: Clinicopathological features of two clusters classified by consensus clustering based on cellular response to IL-4-related gene set in CGGA dataset; Table S2: Clinicopathological features of two gene risk groups in CGGA and TCGA dataset.
