# Supplementary material for: Identification of an IL-4-Related Gene Risk Signature for Malignancy, Prognosis and Immune Phenotype Prediction in Glioma
Source: Brain Sci. 2022 Jan 29;12(2):181. doi: 10.3390/brainsci12020181 (PMC8870251; doi:10.3390/brainsci12020181)
Supplement: Supplementary file 1 [file brainsci-12-00181-s001.zip › Table S1.pdf]

Table S1: Clinicopathological features of two clusters classified by consensus clustering based on cellular response to IL-4-related gene set in CGGA dataset.

**Features**	**Cluster 1 (n=124)**	**Cluster 2 (n=201)**	***P* value**
**Age**			< 0.0001
Median	38	46	
**Gender**			0.3513
Female	51	71	
Male	73	130	
**TCGA subtype**			< 0.0001
Classical	10	64	
Mesenchymal	7	61	
Neural	33	48	
Proneural	74	28	
**WHO Tumor grade**			< 0.0001
II	67	42	
III	30	42	
IV	27	117	
**IDH status**			< 0.0001
Mutation	108	59	
Wildtype	16	142	
**1p/19q status**			< 0.0001
Codeletion	35	20	
Non-codeletion	84	166	
NA	5	15	
