# Supplementary material for: Identification of an IL-4-Related Gene Risk Signature for Malignancy, Prognosis and Immune Phenotype Prediction in Glioma
Source: Brain Sci. 2022 Jan 29;12(2):181. doi: 10.3390/brainsci12020181 (PMC8870251; doi:10.3390/brainsci12020181)
Supplement: Supplementary file 1 [file brainsci-12-00181-s001.zip › Table S2.pdf]

Table S2: Clinicopathological features of two gene-risk groups in CGGA and TCGA dataset.

**Features**	**Training set CGGA RNA-seq cohort (n=325)**			**Validation set TCGA RNA-seq cohort (n=667)**		
	**High-risk group (n=162)**	**Low-risk group (n=163)**	***P* value**	**High-risk group (n=333)**	**Low-risk group (n=334)**	***P* value**
Age			<0.001			<0.001
Median	46.5	39		54	39	
Gender			0.148			0.734
Female	54	68		136	146	
Male	108	95		191	192	
TCGA subtype			<0.001			<0.001
Classical	60	14		85	1	
Mesenchymal	68	0		97	0	
Neural	9	72		31	79	
Proneural	25	77		55	182	
WHO Tumor grade			0.002			<0.001
II	13	96		42	206	
III	34	38		131	131	
IV	115	29		155	1	
IDH status			<0.001			<0.001
Mutation	35	132		98	327	
Wildtype	127	31		225	10	
1p/19q status			<0.001			<0.001
Codeletion	7	48		10	158	
Non-codeletion	140	110		311	181	
