# Supplementary figures and images for: Identification of an IL-4-Related Gene Risk Signature for Malignancy, Prognosis and Immune Phenotype Prediction in Glioma
Source: Brain Sci. 2022 Jan 29;12(2):181. doi: 10.3390/brainsci12020181 (PMC8870251; doi:10.3390/brainsci12020181)

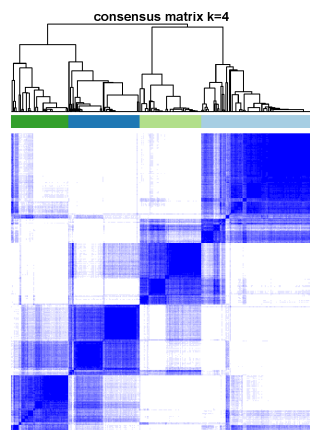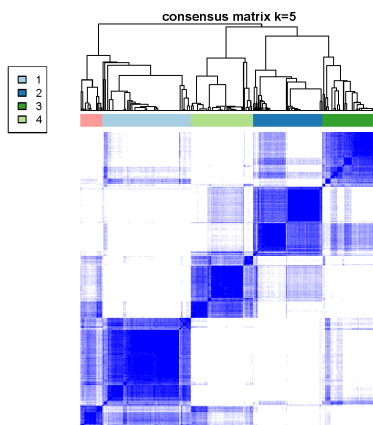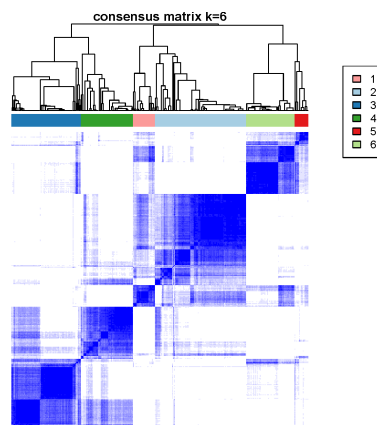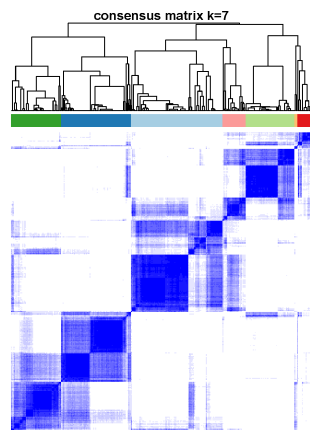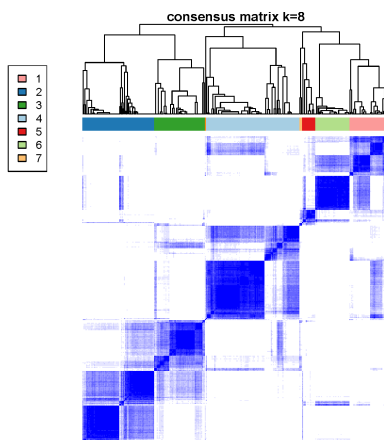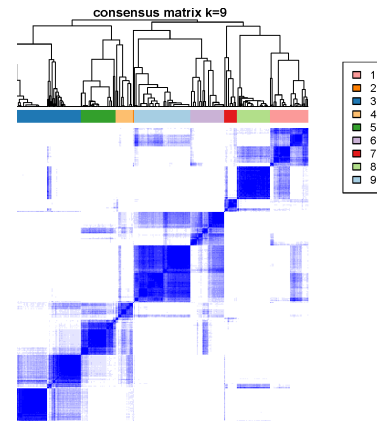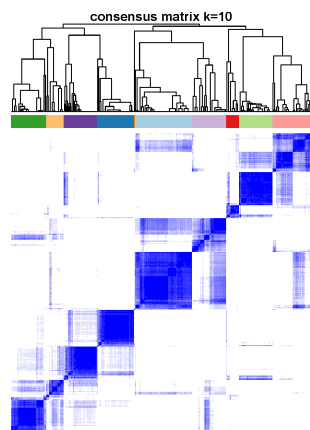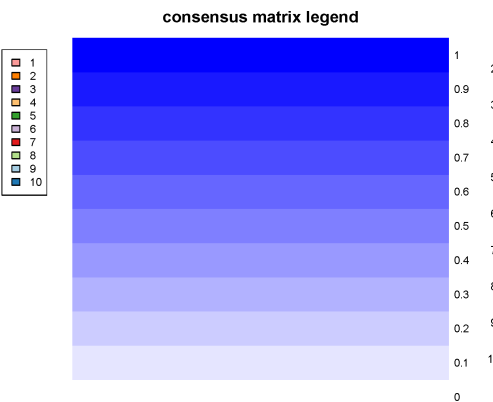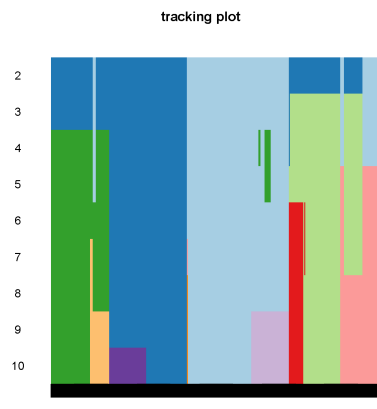

samples

Supplement: Supplementary file 1 [file brainsci-12-00181-s001.zip › Figure S1.pdf]

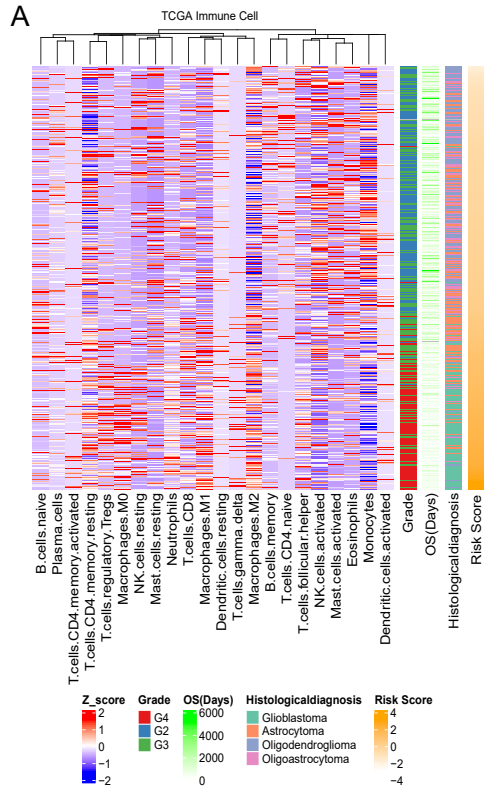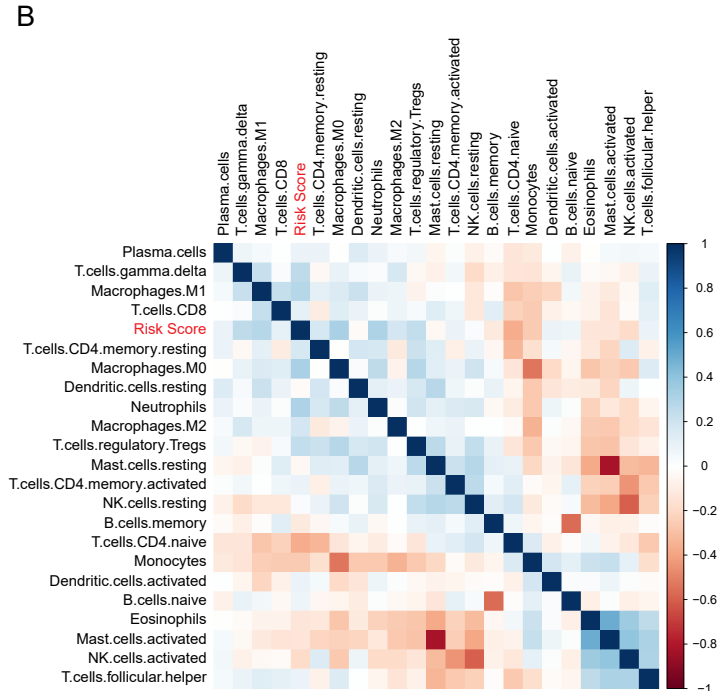

Supplement: Supplementary file 1 [file brainsci-12-00181-s001.zip › Figure S2.pdf]
